# ﻿Revising the diversity within the Dwarf Dog-faced Bat, *Molossopstemminckii* (Chiroptera, Molossidae), with the revalidation of the endangered Molossopsgriseiventer

**DOI:** 10.3897/zookeys.1180.109091

**Published:** 2023-09-25

**Authors:** Héctor E. Ramírez-Chaves, Darwin M. Morales-Martínez, Daniela Martínez-Medina, Paula A. Ossa-López, Fredy A. Rivera-Páez

**Affiliations:** 1 Grupo de Investigación en Genética, Biodiversidad y Manejo de Ecosistemas (GEBIOME), Departamento de Ciencias Biológicas, Facultad de Ciencias Exactas y Naturales, Universidad de Caldas, Calle 65 No. 26-10, Manizales, Caldas 170004, Colombia; 2 Centro de Museos, Museo de Historia Natural, Universidad de Caldas, Calle 65 No 26-10, Manizales, Caldas, Colombia; 3 Museum of Natural Science and Department of Biological Sciences, Louisiana State University, 119 Foster Hall 70803, Baton Rouge, Louisiana, USA; 4 Colecciones Biológicas, Instituto de Investigación de Recursos Biológicos Alexander von Humboldt, Claustro de San Agustín Villa de Leyva, Colombia; 5 Doctorado en Ciencias, Biología, Facultad de Ciencias Exactas y Naturales, Universidad de Caldas, Calle 65 No. 26-10, Manizales, Caldas 170004, Colombia

**Keywords:** Andes, biogeography, endemism, Mammalia, nomenclature, subspecies, taxonomy

## Abstract

The genus *Molossops* includes two monotypic species of insectivore bats distributed in South America: *Molossopsneglectus* and *Molossopstemminckii*. Both can be differentiated, based on sizes, *M.temminckii* being smaller (forearm less than 33 mm). Despite being monotypic, at least two additional subspecies have been described for *M.temminckii*, of which *M.temminckiigriseiventer* from the inter-Andean Valley of the Magdalena River in Colombia might represent a valid taxon. To test the taxonomic status of *M.t.griseiventer*, we reviewed specimens of *M.temminckii* from cis- and trans-Andean localities in Colombia. We used Cytochrome-b and Cytochrome Oxidase I comparisons to test the phylogenetic position of cis- and trans-Andean samples and compared qualitative morphology, morphometric and bioacoustics. Our results show that *M.t.griseiventer* is differentiated from cis-Andean specimens, providing further evidence of its validity at the species level. Furthermore, *M.temminckii* (sensu stricto) is also distributed in Colombia, but both *M.griseiventer* and *M.temminckii* are allopatric, with the Andes acting as a barrier. The specific identity of the specimens from the Caribbean Region of Colombia needs a new evaluation, but our results clearly show that the diversity of *Molossops* is underestimated.

## ﻿Introduction

The genus *Molossops* Peters, 1866, comprises two species distributed in South America: the Rufous Dog-faced bat, *Molossopsneglectus* Williams & Genoways, 1980 and the Dwarf Dog-faced Bat, *M.temminckii* Burmeister, 1854 (Díaz et al. 2021; Mammal Diversity Database 2022). The genus is monophyletic, based on morphological data (Gregorin and Cirranello 2016), but only *M.temminckii* has been included in the molecular phylogenetic reconstructions of the family using one mitochondrial and three nuclear genes of a single specimen from Paraguay (Ammerman et al. 2012).

Species of *Molossops* are characterised by their small size (forearm less than 39 mm), with relatively large tragus, a short and wide antitragus and long and pointed ears that have a flexible fold where they are attached to the head (Eger 2008). *Molossopsneglectus* and *M.temminckii* are currently considered monotypic (Eger 2008). *Molossopsneglectus* is the rarest species and is only known from a few localities in Colombia, Venezuela, Guyana, Suriname, French Guiana, Peru, Brazil and Argentina. In contrast, *M.temminckii* has a broader distribution, with records in Colombia, Venezuela, Guyana, northern Ecuador, Peru, Brazil (type locality at “Lagoa Santa,” Minas Gerais), Bolivia, Paraguay, Argentina and Uruguay (Gamboa Alurralde and Díaz 2019; Mammal Diversity Database 2022) with a huge gap between northern South American populations and those from the south of the Amazonia.

Despite Eger (2008) treating the species as monotypic, there are two additional described subspecies: *M.t.sylvia* Thomas, 1924, with type locality at “Goya”, Corrientes, Argentina; and *M.t.griseiventer* Sanborn, 1941, with type locality “Espinal, west of Magdalena River on the plains of Tolima, Colombia”, tentatively endemic to the inter-Andean Valley of the Magdalena River of Colombia. *Molossopst.griseiventer* exhibits morphometric differences compared to the nominal population, although, due to the limited samples, its subspecific status was not fully supported (Eger 2008; Gamboa Alurralde and Díaz 2019). In recent years, Chacón-Pacheco et al. (2021) confirmed that individuals of *M.t.griseiventer* are larger than specimens from the Caribbean and Orinoco Llanos Regions in Colombia and *M.griseiventer* was elevated to species level, based on karyotype descriptions of specimens collected in cis-Andean localities in Venezuela (Volleth et al. 2023), far from and separated from the type locality for the Oriental (Eastern) Cordillera of Colombia. However, no other morphological and molecular data have been used to validate the specific status. Here, we assess the cryptic diversity and the validity of *M.t.griseiventer* reflected by the morphometric variation found in previous works (Eger 2008; Chacón-Pacheco et al. 2021) using mitochondrial, morphological and acoustic data. We also evaluated if the morphologic variation is associated with geographical barriers in northern South America.

## ﻿Materials and methods

To explore the cryptic diversity within *M.temminckii*, we collected 18 specimens in both sides of the Andes in Colombia in contrasting areas: the inter-Andean Valley of the Magdalena River (close to the type locality of *M.t.griseiventer*) and the Orinoco Llanos Region (*M.temminckii*). The specimens were deposited at the Instituto de Ciencias Naturales, Universidad Nacional de Colombia (**ICN**) and the Colección de Mamíferos, Museo de Historia Natural of the Universidad de Caldass (**MHN-UCa**). We identified the specimens as belonging to *Molossops*, based on the following traits: tips of ears elongated and pointed with inner margins not arising from the same point, but joined to head by a flexible fold; no granulations on the forearm; the skull flattened; one upper premolar; upper incisors in contact with each other and projecting forward; palate distinctly domed and anterior palate not emarginate; basisphenoid pits clearly developed; lacrimal processes well developed; lacrimal width of rostrum considerably greater than postorbital constriction; one lower incisor in each ramus; third commissure of M3 well developed, about 1/2 the length of the second; the second phalanx of digits III and IV equal to or longer than first (Eger 2008). We also explored discrete traits suggested in literature as unique for *M.t.griseiventer*, such as colouration and size of the patch of the throat and colouration of the venter (Sanborn 1941).

We obtained nine cranial and external measurements including the length of the forearm (FA), greatest length of the skull (GLS), condylo-basal length (CBL), breadth of the braincase (BB), postorbital constriction (POC), width across the last upper molars (M-M), zygomatic breadth (ZB), maxillary tooth length (C-M^3^) and mastoid breadth (MB). To explore the morphometric variation within cis- and trans-Andean populations in Colombia, we performed Principal Component Analyses (PCA) using only seven cranial measurements (GLS, CBL, POC, BB, C-M^3^, MB, M-M). We computed the analyses using the covariance matrix to preserve the information about the relative scale amongst variables using the statistical package PAST version 2.2 (Hammer et al. 2001). We also obtained discriminant analyses and correct classification rates for pairwise comparisons amongst *M.t.griseiventer* and specimens of *M.temminckii* from eastern Colombia and localities in the Southern Cone of South America.

To compare genetic affinities of the cis- and trans-Andean specimens of *M.temminckii*, we extracted genomic DNA from muscle tissues preserved in 96% ethanol of five specimens. Following the manufacturer’s protocol, DNA was extracted with a Wizard Genomic DNA Purification kit (Promega Corporation). We performed amplification of a fragment of the mitochondrial gene Cytochrome-b (Cyt-b) using the primers LGL765F (Bickham et al. 1995) and LGL766R (Bickham et al. 2004) and the reaction volumes and the amplification conditions detailed in Ramírez-Chaves et al. (2021a). We checked for discontinuities and stop codons for all the obtained sequences using the ExPASy translate web tool (https://web.expasy.org/translate/). We gathered homologous sequences of Neotropical species of *Molossops* from GenBank including seven Cyt-b sequences of *M.temminckii*. We also analysed the genetic affinities in the mitochondrial gene Cytochrome Oxidase I (COI). For that gene, we retrieved sequences deposited in GenBank and Bold Systems platforms, including a sequence of *M.t.griseiventer* from Caucasia in the inter-Andean Valley of the Cauca River in the Department of Antioquia, Colombia and 23 sequences of *M.neglectus* (16 sequences) and *M.temminckii* (7 sequences) species. GenBank and Bold Systems accessions, museum vouchers and localities of the analysed sequences are shown in Appendix 1.

We inferred phylogenetic trees for each gene using Bayesian Inference in MrBayes 3.2.6 (Ronquist et al. 2012). We ran two independent replicates of the Metropolis coupled chain Monte Carlo analysis for 10×10^6^ generations with trees sampled every 1,000 generations. Convergence was inspected in the programme Tracer 1.6 (Rambaut et al. 2014). We discarded 25% of the samples in each run as burn-in and combined the remaining samples to estimate tree topology, the mean likelihood and posterior probabilities. We used different outgroup sets for each gene because of the different availability of sequences. The species *Cynomopsplanirostris*, *Eumopsauripendulus*, *Nyctinomopslaticaudatus* and *Tadaridateniotis* were used as outgroups for the COI gene and *Cynomopsabrasus*, *E.auripendulus*, *Nyctinomopsaurispinosus* and *T.teniotis* for Cyt-b analyses. Besides, we estimated genetic distance values for Cyt-b and COI using the p-distance method in the software MEGA X (Kumar et al. 2018). We analysed the haplotype network for Cyt-b and COI genes to assess non-bifurcated similarities. We trimmed all sequences to equal lengths and removed the sequences containing ambiguities (Cyt-b = 1071 bp, COI = 650 bp). Posteriorly, we built each haplotype network using the TCS parsimony algorithm (Clement et al. 2002) as implemented in PopArt (Population Analysis with Reticulate Trees) 1.7 software.

Finally, we examined acoustic variables in the echolocation calls of *M.temminckii* from southern South America and *M.t.griseiventer* from the Magdalena River Basin to explore their differences. Recordings of *M.t.griseiventer* were obtained from two different localities in the Magdalena River Basin using transects (AnaBat Walkabout, Titley Scientific) and passive acoustic monitoring (AnaBat Swift, Titley Scientific) methods. Our data were compared with available information on the species from Brazil (Oliveira et al. 2018). Spectrograms and oscillograms of echolocation call sequences were displayed simultaneously using Raven Pro 1.6 software (K. Lisa Yang Center for Conservation Bioacoustics 2023) from 512 consecutive fast Fourier transforms with an 85% overlap and visualised on a Hamming-type window. For each pulse in the sequences analysed, we manually measured the following parameters: 1) call duration (measured in ms from the start to the end of the pulse), 2) peak frequency (corresponding to maximal intensity in the power spectrum), 3) maximum, 4) and minimum frequency and, finally, 5) the bandwidth (measured as the absolute difference between Fmax and Fmin). Recordings of *M.t.griseiventer* are available at the Environmental Sound Collection – ‘Mauricio Álvarez-Rebolledo’ (Colección de Sonidos Ambientales IAvH-CSA) at the Instituto Humboldt in Colombia.

### ﻿Species delimitation

Considering the potential presence of cryptic diversity, we expected that *M.t.griseiventer* from Magdalena inter-Andean Valley will constitute a monophyletic group with more than 3% divergence with the other populations following the Genetic Species Concept (Bradley and Baker 2001) and supported by morphological and acoustic differences.

## ﻿Results

### ﻿Morphological analysis

**Figure 1.**
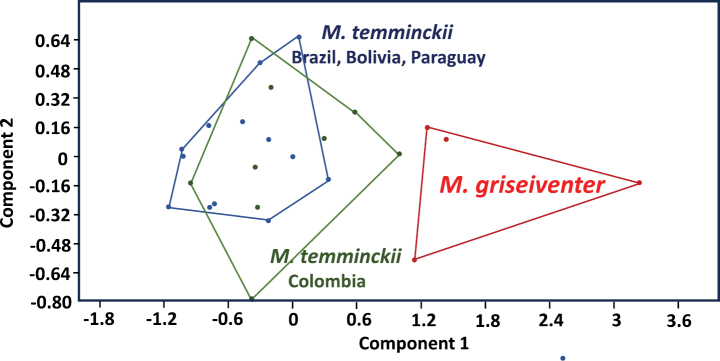
PCA plot (first two PCs) of small species of *Molossops* including *M.temminckii* from southern South America, specimens of *M.temminckii* from cis-Andean Colombia (Orinoco Llanos) and *M.griseiventer* from the Magdalena River Basin of Colombia. Note the spatially disjointed distribution of the two groups assigned to *M.temminckii* and *M.griseiventer*.

Cis- and trans-Andean specimens of *Molossopstemminckii* are small (e.g. FA: 28.0–32.9 mm; GLS: 13.2–15.3 mm) and exhibited the diagnostic traits of the genus. We found a set of qualitative morphology and morphometric characters that distinguish *M.t.griseiventer* from *M.temminckii*. Qualitative morphological characters are detailed in the re-description of the species below. The PCA analysis showed size differences, the first two components using seven cranial measurements (Table 1) of 27 specimens account for 87.25% (PC1 = 77.97%, PC2 = 9.28%) of the variation and these are related to the CBL and the GLS measurements, both in the PC1 and PC2 (Fig. 1). The discriminant analysis of the skull measurements showed a percentage of 100% (F = 7.735; P < 0.001) between *M.temminckii* (from eastern Colombia and the Southern Cone) vs. *M.t.griseiventer*, and 68.18% (F = 0.803; P = 0.598) of correctly-classified individuals between *M.temminckii* from eastern Colombia vs. Southern Cone specimens (Brazil, Bolivia, Paraguay). However, only four specimens of *M.t.griseiventer* were included in the analysis.

**Table 1.** Principal Component Analyses results for seven cranial measurements of 27 specimens of small *Molossops*. CBL and the GLS contributed the most in the PC1 and PC2.

PC	Eigenvalue	% variance
1	0.960491	77.976
2	0.114265	9.2764
3	0.063141	5.126
4	0.055469	4.5032
5	0.018439	1.497
6	0.012043	0.97771
7	0.007937	0.64431

### ﻿Molecular analyses

**Figure 2.**
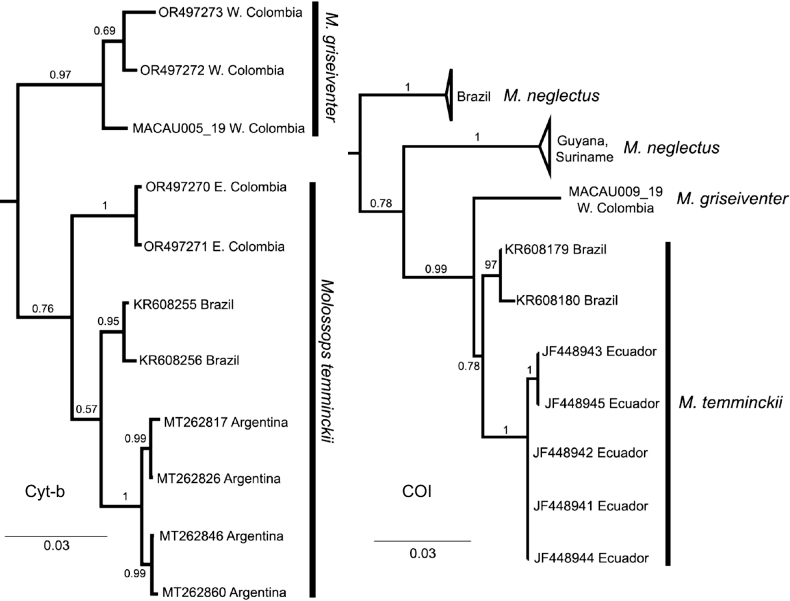
Phylograms of Cyt-b and COI mitochondrial genes of *Molossops*. Branch values indicate the posterior probability of each clade obtained through Bayesian Inference.

Sequence alignments for Cyt-b and COI sequences were unequivocal and without internal stop codons. The Cyt-b gene sequences of *M.t.griseiventer* made up a highly-supported monophyletic group (pp = 0.97), sister of a low-supported clade of *M.temminckii* from eastern Colombia, Argentina and Brazil (pp = 0.76; Fig. 2). The mean genetic distances from *M.t.griseiventer* with respect to different populations of *M.temminckii* were greater than 4% (from 4.15% Brazil to 4.90% Argentina). The mean genetic distance between *M.t.griseiventer* and *M.temminckii* from the cis-Andean populations of Colombia was 4.48% (Table 2). The genetic distances between specimens of *M.temminckii* from cis-Andean populations of Colombia and those populations from Argentina and Brazil were less than 3% (2.75% from Brazil and 2.80% from Argentina; Table 2).

**Table 2.** Cytochrome b (Cyt-b) and Cytochrome Oxidase I (COI) distances amongst *Molossops* taxa. W: Western Colombia (trans-Andean). E. Eastern Colombia (cis-Andean).

COI gene	1	2	3	4	5
1. *M.neglectus* Guyana and Suriname	0.00				
2. *M.neglectus* Brazil	6.51	0.00			
3. *M.temminckii* Ecuador	6.21	6.70	0.00		
4. *M.temminckii* Brazil	6.01	5.83	1.89	0.00	
5. *M.griseiventer* W. Colombia	6.22	6.73	3.78	3.46	NA
**Cyt-b gene**	**1**	**2**	**3**	**4**	
1. *M.temminckii* E. Colombia	0.22				
2. *M.temminckii* Argentina	2.80	0.29			
3. *M.temminckii* Brazil	2.75	2.01	0.00		
4. *M.griseiventer* W. Colombia	4.48	4.90	4.15	0.66	

The COI gene recovered the only sequence of *M.t.griseiventer* sister to a low-supported clade of *M.temminckii* (pp = 0.78; Fig. 2). Genetic distances of *M.t.griseiventer* in the COI were greater than 3% and ranged from 3.46% from Ecuador, 3.97% from Brazil and greater than 6%, respectively, than both *M.neglectus* clades (Table 2).

**Figure 3.**
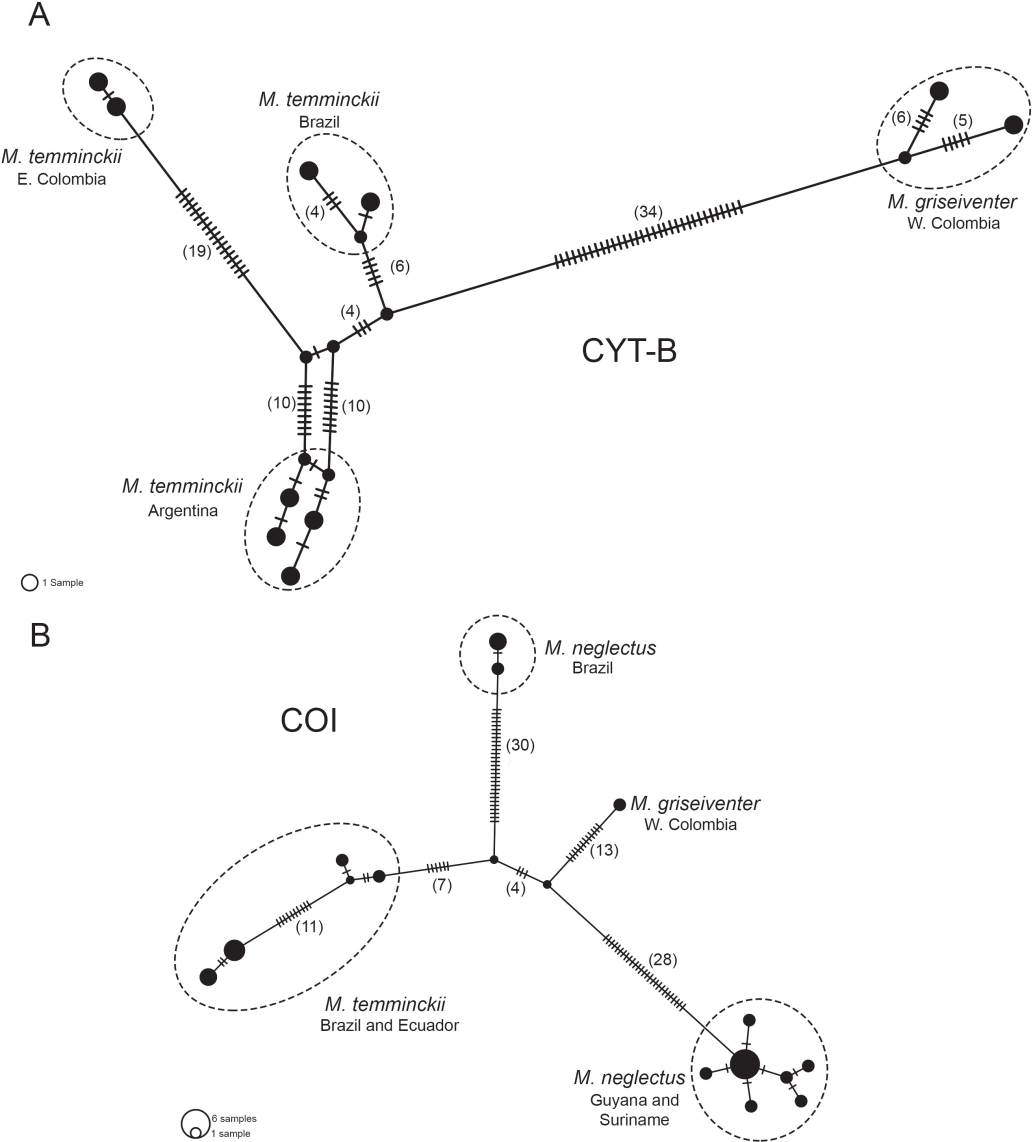
Haplotype networks of Cyt-b (**A**) and COI (**B**) genes constructed by the TCS parsimony algorithm. Nodes are proportional to the frequency of individuals carrying the allele. Numbers in parentheses represent the number of mutational steps > 3.

The haplotype networks for the COI and the Cyt-b genes showed that *M.t.griseiventer* conformed independent haplotypes in both genes compared with *M.temminckii*. In the Cyt-b haplotype network, *M.t.griseiventer* formed two haplotypes separated between 5 and 6 mutational steps. These haplotypes are separated by more than 40 mutational steps of the haplotypes of *M.temminckii*, which are separated between two to 28 mutational steps (Fig. 3). In the COI gene, the haplotype of *M.t.griseiventer* is unique and it is separated by more than 31 mutational steps from *M.temminckii* and two clusters of *M.neglectus* (Fig. 3).

### ﻿Acoustic analyses

**Figure 4.**
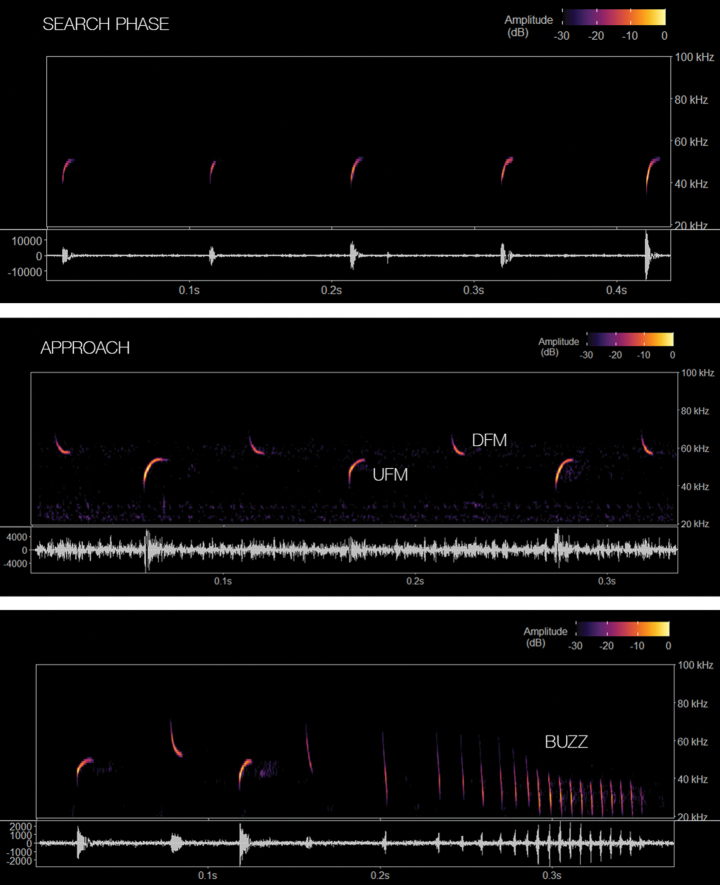
Echolocation behaviour of *M.griseiventer* in a sequence of pulses from the search phase through approach and feeding buzz phases.

We analysed 174 pulses from 10 call sequences of *M.t.griseiventer* (Fig. 4). The echolocation behaviour of *M.temminckii* sets it apart from other species within the Molossidae because it employs two distinct types of signals: upward frequency modulated (UFM) signals and downward frequency modulated (DFM) signals (Guillén-Servent and Ibáñez 2007). When comparing the reported results for *M.temminckii* from southern South America, notable differences were observed across all acoustic variables (Table 3). The UFM search phase pulses from Brazil (on manual release) exhibited shorter durations, higher peak frequencies and narrower bandwidths than those of *M.t.griseiventer*. Further examination of the remaining phases revealed distinct variations in the DFM signals, particularly in duration, minimum and peak frequencies. The signals from the Magdalena River Basin exhibited considerably higher duration and frequencies (Table 3).

**Table 3.** Parameters of free flying bat echolocation pulses of *M.griseiventer* from the Magdalena River Basin of Colombia and parameters of echolocation call pulses of *M.temminckii* from southern South America (Cerrado biome, Federal District, Brazil) using different methodologies. Mean ± Standard Deviation (X ± SD), ms = milliseconds, kHz = kilohertz, n = number echolocation pulses and N = number sequences. DFM = downward frequency-modulated, UFM = upward frequency-modulated.

	This study	Brazil: Oliveira et al. (2018)			
	Free flying	Manual release	Zip-line	Corridor	Tent
**UFM calls**					
**n/N**	137/10	142	353	30	202
**Duration [ms] (X ± SD)**	9.17 ± 1.0	4.11 ± 0.11	5.01 ± 0.07	3.99 ± 0.35	3.45 ± 0.07
**Min freq [kHz] (X ± SD)**	39.62 ± 2.7	43.98 ± 0.20	45.59 ± 0.14	44.06 ± 0.43	47.36 ± 0.16
**Max freq [kHz] (X ± SD)**	53.43 ± 1.5	51.74 ± 0.14	52.51 ± 0.05	52.92 ± 0.21	53.56 ± 0.08
**Peak freq [kHz] (X ± SD)**	46.07 ± 2.5	49.38 ± 0.15	50.95 ± 0.06	51.30 ± 0.18	51.91 ± 0.09
**Bandwidth [kHz] (X ± SD)**	13.8 ± 2.5	7.76 ± 0.12	6.92 ± 0.12	8.86 ± 0.41	6.20 ± 0.13
**DFM calls**					
**n/N**	37/7	57	117	321	242
**Duration [ms] (X ± SD)**	7.28 ± 1.3	3.23 ± 0.15	2.47 ± 0.08	2.05 ± 0.02	1.90 ± 0.04
**Min freq [kHz] (X ± SD)**	54.67 ± 2.0	43.31 ± 0.51	46.74 ± 0.48	40.08 ± 0.27	44.26 ± 0.43
**Max freq [kHz] (X ± SD)**	77.71 ± 9.3	58.82 ± 0.63	64.50 ± 0.37	66.30 ± 0.21	68.01 ± 0.29
**Peak freq [kHz] (X ± SD)**	57.81 ± 1.27	45.62 ± 0.81	54.20 ± 0.20	52.99 ± 0.22	55.70 ± 0.26
**Bandwidth [kHz] (X ± SD)**	23.04 ± 10.7	16.51 ± 0.45	17.75 ± 0.69	26.21 ± 0.34	23.75 ± 0.51

### ﻿Taxonomic remarks

We recognise *M.griseiventer* from the Magdalena River inter-Andean Valley as a valid species, based on genetic, acoustic and morphological evidence. Genetically, *M.griseiventer* conformed monophyletic groups in two genes; they have genetic distances greater than 3% with respect to populations of *M.temminckii* and independent haplotypes separated from *M.temminckii* populations.

## ﻿Discussion

Our analyses clearly showed the validity at the species level of *M.griseiventer*, based on morphologic and genetic evidence supporting previous claims of morphometric differentiation of this taxon when compared with *M.temminckii* (Eger 2008). While differences in all acoustic variables for both UFM and DFM signals were observed, further analyses are necessary to determine the statistical significance of these differences. Notably, variations were evident across all recording contexts used in Brazil (Oliveira et al. 2018). However, particular emphasis should be placed on the recordings captured during manual release, as this context closely resembles free-flying conditions.

Cryptic diversity within *Molossops* was previously suggested, based on morphometric comparisons (Eger 2008; Chacón-Pacheco et al. 2021) and the diversity within the genus may still be under-described. For example, Volleth et al. (2023) argued that differences between the karyotypes of specimens from cis-Andean Colombia and Venezuela and those from Brazil and published morphometric data (i.e. Chacón-Pacheco et al. 2021) are enough to elevate the subspecies *M.t.griseiventer* to species rank. However, although we provide evidence of the validity of *M.griseiventer* as a full species here, the conclusion of Volleth et al. (2023) is incorrect, but supports the presence of possible additional cryptic diversity. Volleth et al. (2023) linked the karyomorphs from the Orinoco Region with *M.t.griseiventer* even though these are distant from the type locality and are separated by the Cordillera Oriental of Colombia. The Orinoco Llanos´ karyotypes correspond to the populations of *M.temminckii* of northern South America and the cranial and external measurements provided for these specimens fall within the range of the specimens from eastern Colombia (Table 4), being smaller than those of *M.griseiventer*. Consequently, the specimens of *M.temminckii* from eastern Colombia and western Venezuela for which the karyomorph is 2n = 42, FNa = 56–60 (Gardner 1977; Volleth et al. 2023) could represent a different species. We found that cis-Andean specimens of Colombia and Ecuador formed monophyletic groups in both COI and Cyt-b genes. Nevertheless, we did not explore morphometric, morphologic or acoustic differences and the values of genetic distances are lower than 3% in both genes. Therefore, although these populations could represent a distinct undescribed taxon, we do not have the evidence to describe them. We also highlight the presence of at least two distinct species within *M.neglectus*, one from Brazil and one from Suriname and Guyana recovered as two different monophyletic groups in our genetic analysis.

**Table 4.** Measurements of small *Molossops* of South America. The measurements of the holotype are taken from Sanborn (1941). Values indicate the mean (range) and n. SMF: Senckenberg Museum/Frankfurt, Germany. The specimens from eastern Colombia are deposited at the MHN-UCa (MHN-UCa 2274-2281; 2297-2300; 3706-3707, 3715). Specimens from southern South America are deposited at the FMNH (Brazil: FMNH 20890. Bolivia: FMNH 115814-115819. Paraguay: FMNH 44100-44101, 44263, 48773-48775, 53928, 54399-54400, 66232, 63863).

	*M.griseiventer* Holotype FMNH 51727	*M.griseiventer* Paratype FMNH 51726	*M.griseiventer*MHN-UCa 4220, ICN 25759, ICN 25760	*M.temminckii* Eastern Colombia	*M.temminckii* southern South America	*M.temminckii* Venezuela (Volleth et al. 2023) SMF 72269
Forearm	31.9	30.7	(31.3–32.9) 3	29.44 (28.66–31.10) 12	30.12 (27.70–32.00) 23	29
Greatest length of skull	15.3	14.60	14.63 (14.04–15.34) 4	13.57 (13.18–14.02) 8	13.48 (12.99–14.40) 13	13.8
Condylo-basal length	13.8	14.18	14.62 (14.16–15.65) 4	13.27 (12.78–13.88) 8	12.96 (12.55–13.59) 13	12.5
Postorbital constriction	4.3	4.23	4.18 (3.97–4.34) 4	4.02 (3.74–4.38) 8	3.87 (3.66–4.05) 13	-
Zygomatic width	9.6	broken		9.35 (8.92–9.56) 6		9.0
Mastoid width	8.9	9.06	9.06 (8.66–9.70) 4	8.69 (8.11–9.25) 8	8.41 (7394–9.08) 13	8.25
Breadth of braincase	7.4	7.53	7.44 (7.29–7.65) 4	7.31 (6.84–7.57) 8	7.20 (6.82–7.46) 13	-
Upper tooth-row length	5.7	5.78	5.75 (5.58–5.97) 4	5.42 (5.25–5.65) 8	5.27 (5.00–5.50) 13	5.5
Width across molars	6.8	6.77	6.92 (6.67–7.23) 4	6.53 (5.67–6.92) 8	6.45 (6.09–6.82) 13	6.15

Our review also increases the number of *Molossops* species to three and highlights the role of the Andes in the diversification of the genus, as has been suggested for other bat species (Patterson et al. 2012). With these findings, the number of molossid species in Colombia reaches 30 species (Ramírez-Chaves et al. 2021b), a richness like other highly diverse countries in South America, such as Brazil (32 species; Garbino et al. (2022)) and Peru (31 species; Velazco (2021)). Furthermore, the number of bat species in Colombia reaches 220 (see Ramírez-Chaves et al. (2021b); Esquivel et al. (2022)).

The distribution limits of *M.griseiventer* are still unclear. So far, *M.griseiventer* is restricted to Colombia between Antioquia and Huila Departments, containing the first genus records for the Department of Caldas (Ramírez-Chaves et al. 2021c). However, the identity of specimens from the Caribbean Region of Colombia (Chacón-Pacheco et al. 2021), from Cucuta (Royal Ontario Museum ROM 84999) and the Biogeographic Chocó (Bahía Solano: ROM 69533) are still in need of a taxonomic evaluation, these potentially being extensions of the distribution of the species. To contribute to the specific differentiation of *M.griseiventer*, we present amended systematic information for the species:

### ﻿Order Chiroptera

**Family Molossidae P. Gervais, 1856**

**Genus *Molossops* W. Peters, 1866**

*Molossopsgriseiventer* Sanborn, 1941. New rank.

*Molossopstemminckiigriseiventer* Sanborn, 1941:385. Type locality “Espinal, west of Magdalena River on the plains of Tolima, Colombia.”

*Molossopstemminckii*: Chacón-Pacheco et al. 2021. Part. Not *Molossopstemminckii* (Burmeister 1854: 72).

*Molossopsgriseiventer*: Volleth et al. 2023:3. Part. First use of the current combination.

**Type material. *Holotype***: Field Museum of Natural History (FMNH 51727), adult female in alcohol with skull removed. Collected on 21 September 1940 by Brother Nicéforo María (Sanborn 1941).

**Type locality.** “Espinal, west of Magdalena River on plains of Tolima, Colombia.” The type locality is currently located in the Department of Tolima.

**Figure 5.**
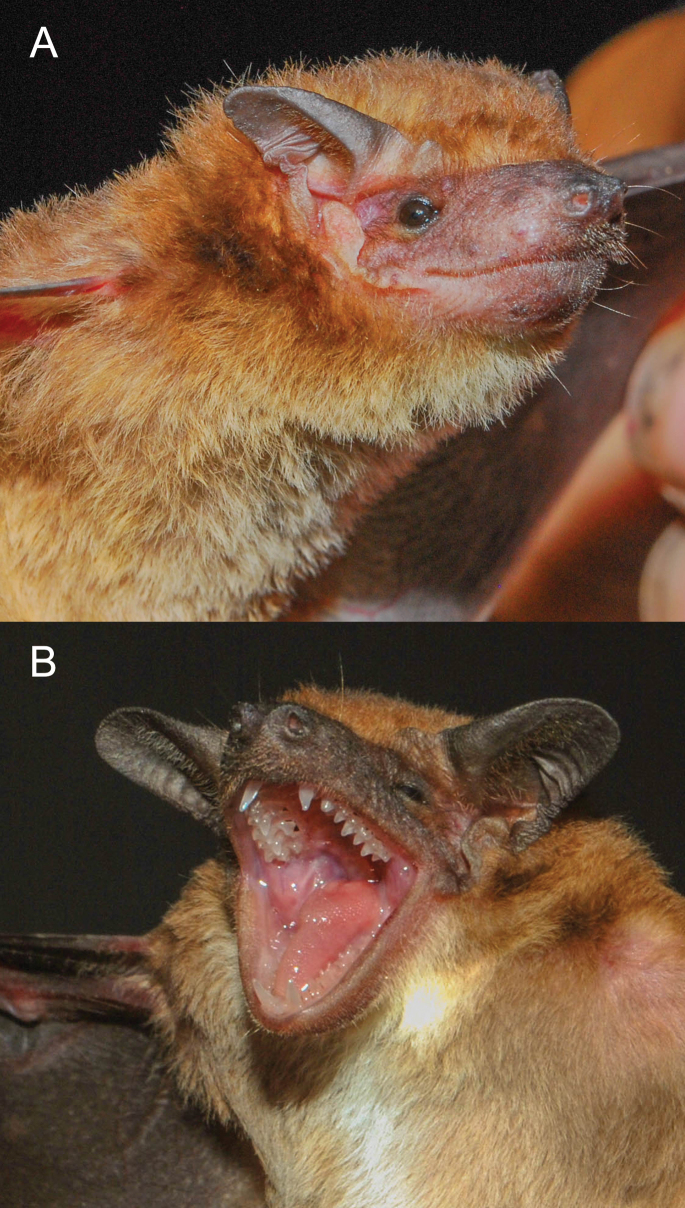
Details specimens of dwarf dog-faced bat (*Molossops*) **A***Molossopstemminckii***B***Molossopsgriseiventer*. Note the elongated and pointed tips of ears and the smooth lips in both specimens, the differences in ears and rostrum colouration being darker in *M.griseiventer*, the white gular patch in *M.griseiventer* that extends into the chest and the differences in the antitragus being more robust and rounded in *M.griseiventer*.

**Figure 6.**
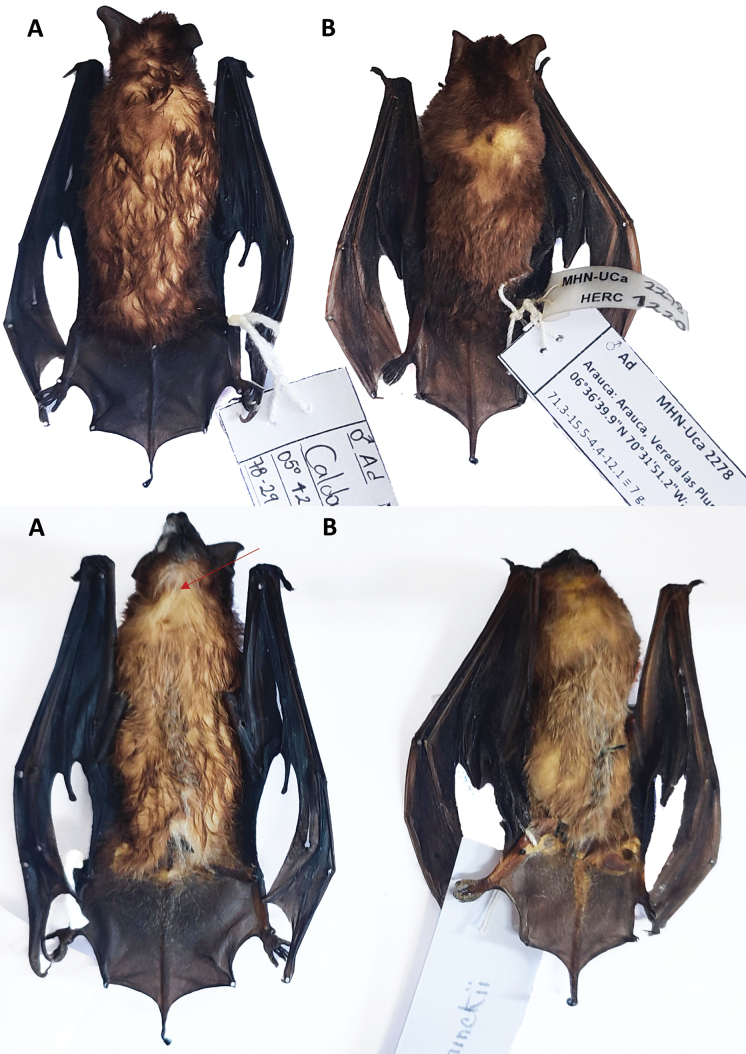
Details of the skin of males **A***Molossopsgriseiventer* and **B***Molossopstemminckii*, from Colombia. Note the darker colouration of the membranes in *M.griseiventer*. Red arrow shows the lighter ventral patch in the throat.

**Figure 7.**
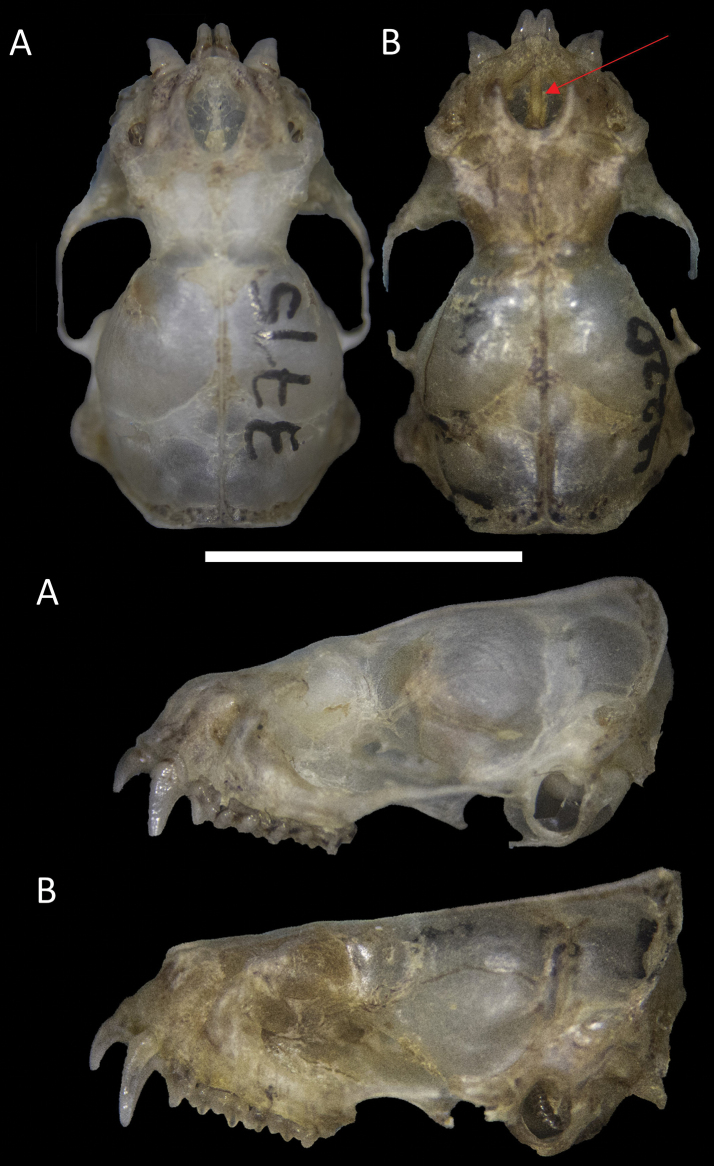
Details of the skull of **A***Molossopstemminckii* and **B***Molossopsgriseiventer*, from Colombia. Red arrow shows the marked nasal septum in *M.griseiventer*. Note the differences in the size of the upper incisors and the shape of the skull in lateral view. Scale bar: 10 mm.

**Emended diagnosis.** A small molossid bat (FA: 30.7–31.9 mm; weight: 7 g) with brownish dorsal colouration. Ventrally, it exhibits a white patch around 9 × 7 mm on the throat. Externally, the tips of ears are elongated and pointed with inner margins not arising from the same, but joined to the head by a flexible fold; lips smooth, lacking evident facial papillae (Fig. 5). The nose, ears and membranes are blackish (Fig. 6). The skull is small (GLS: 14.04–15.34 mm) and flattened (Fig. 7). The inner incisors are long, reaching almost half of the length of the canines. The postorbital constriction is well marked (3.97–4.34 mm); the sagittal crest is present, but low.

**Figure 8.**
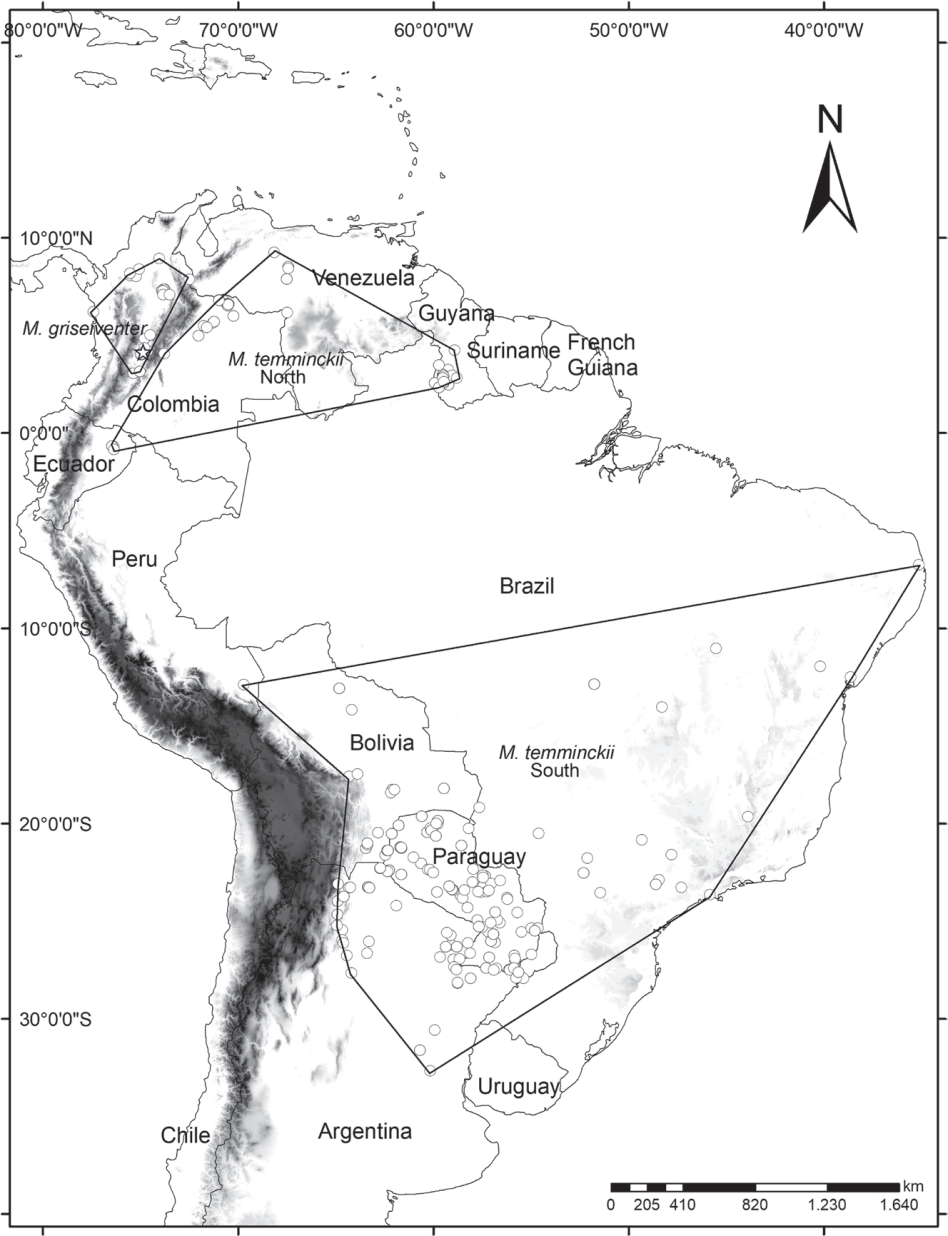
Distribution of *Molossopsgriseiventer* and *Molossopstemminckii* in South America. We detailed the allopatric distribution of two cis-Andean populations of *Molossopstemminckii* from “North” and “South” South America. The star represents the type locality of *Molossopsgriseiventer* in the inter-Andean Valley of the Magdalena River. Trans-Andean records are attributed to *M.griseiventer*.

**Distribution.***M.griseiventer* is distributed in the inter-Andean Valleys of the lower Cauca and the Magdalena River Basin. All the locality records are trans-Andean and under 900 m a.s.l. (Fig. 8). The species is only confirmed from localities in the Departments of Antioquia, Cundinamarca, Huila, Caldas, Santander and Tolima, but additional records from the Magdalena Valley need confirmation.

**Comparisons.***M.neglectus* is larger (forearm > 36 mm) than *M.griseiventer* and *M.temminckii*. *Molossopsgriseiventer* is similar to *M.temminckii* (Table 4), but can be differentiated by a set of characters. Externally, *M.griseiventer* has darker colouration of the ears, nose and membranes than *M.temminckii* (Fig. 7). General colouration varies in *M.temminckii*. The largest series revised from the cis-Andean lowlands of the Orinoco Region of Colombia shows variation in the dorsal fur colouration ranging from light to yellowish-brown to brown and a clear monotonous venter. *M.griseiventer* has a dark brown dorsal colouration, but the venter shows a large white patch on the throat (Fig. 6). The antitragus of live individuals is more elongated in *M.temminckii* than in *M.griseiventer*, which is more rounded; that characteristic is lost in dry specimens. The lips of both *M.griseiventer* and *M.temminckii* from Colombia lack facial papillae (present in *M.temminckii* from Argentina and Brazil; Gregorin and Cirranello (2016); Gamboa Alurralde and Díaz (2019)). *M.griseiventer* appears, in general, larger in external and cranial measurements (Table 4), such as the forearm (31.3–32.9 mm versus 28.9–32.5 mm) and the post-orbital constriction (3.97–4.34 mm versus 3.4–4.1 mm). The skull is more flattened and larger, with a more defined nasal septum in *M.griseiventer* (Fig. 7) than in *M.temminckii* from Colombia (but similar in shape to the skull of *M.temminckii* from Argentina, see: Gamboa Alurralde and Díaz (2019)).

### ﻿Conservation priorities of *Molossopsgriseiventer*

*Molossopsgriseiventer*, to date, is endemic to Colombia and restricted to a few localities of the lowland inter-Andean Valleys of the northern part of the lower Cauca and the Magdalena Rivers in dry forests. These forests have serious conservation threats; it is estimated that less than 10% of the original dry forest in Colombia remains (González-M et al. 2018) and these patches of forest are threatened by deforestation for cattle-ranching, oil palm, as well as the development of hydroelectric projects in the Magdalena River Basin (Angarita et al. 2018). Estimations of the extent of occurrence and area of occupancy using the minimum convex polygon (based on user-defined cell width of 2 km; Bachman et al. 2011) are 96,296 and 124 km^2^, respectively. Considering restricted distribution and deforestation pressure, the species could be endangered and require an urgent evaluation of its threat category. Furthermore, the knowledge about the mammal diversity of the inter-Andean dry forest of Colombia is highly limited. Beyond the endemic dry forest species *M.griseiventer* recognised here, recently another molossid bat, *Cynomopskuizha* (Arenas-Viveros et al. 2021), was described from the Cauca river dry forests; and there is evidence of almost two possible restricted taxa, the bat *Glossophagalongirostrisreclusa* (Webster and Handley 1986) and the populations of *Rhipidomyslatimanus* distributed in the Cordillera Oriental at the Department of Huila (Tribe 2015), that are in need of taxonomic revision. This highlights not only the precarious conservation status of the forest, but also that the mammalian fauna of these forests could be new and an urgent assessment of the conservation priorities for mammals is required.

## ﻿Additional information

### Author ORCIDs

Héctor E. Ramírez-Chaves https://orcid.org/0000-0002-2454-9482

Darwin M. Morales-Martínez https://orcid.org/0000-0001-5786-4107

Daniela Martínez-Medina https://orcid.org/0000-0002-9431-8399

Fredy A. Rivera-Páez https://orcid.org/0000-0001-8048-5818

Paula A. Ossa-López https://orcid.org/0000-0002-9079-4988

### Data availability

All of the data that support the findings of this study are available in the main text.

## ﻿Appendix 1

**Table A1.** Sequences used for phylogenetic inferences. New sequences are shown in bold. * Sequences obtained from Bold Systems.

Species	Locality	Museum voucher	Cytb	COI
*Molossopsneglectus*	Brazil, Parana			MG182653
	Brazil, Sao Paulo			OM849245
	Brazil, Sao Paulo			OM863950
	Brazil, Sao Paulo			OM850171
	Guyana, Potaro-Siparuni	ROM 108484		EF080456
	Guyana, Potaro-Siparuni	ROM 108446		EF080457
	Guyana, Potaro-Siparuni	ROM 108447		EF080458
	Guyana, Potaro-Siparuni	ROM 108481		EF080459
	Guyana, Potaro-Siparuni	ROM 108482		EF080460
	Guyana, Potaro-Siparuni	ROM 108483		EF080461
	Suriname, Nickerie	ROM 117107		JF447678
	Suriname, Nickerie	ROM 117036		JF447679
	Guyana, Potaro-Siparuni	ROM 119805		JF459204
	Guyana, Potaro-Siparuni	ROM 108424		JN312045
	Guyana, Potaro-Siparuni	ROM 108425		JN312046
	Suriname, Sipaliwini	ROM 117465		EU096787
*Molossopstemminckii*	Argentina, Las Colonias		MT262817	
	Argentina, Las Colonias		MT262826	
	Argentina, La Capital		MT262846	
	Argentina, Concordia	MFA-ZV-M 1371	MT262860	
	Brazil, Minas Gerais	M451	KR608255	KR608179
	Brazil, Minas Gerais	M452	KR608256	KR608180
***Molossopstemminckii***	**Colombia, Arauca**	**MHN-UCa 2275**	**OR497270**	
	**Colombia, Arauca**	**MHN-UCa 2280**	**OR497271**	
*Molossopstemminckii*	Ecuador, Napo	ROM 105357		JF448941
	Ecuador, Napo	ROM 105876		JF448942
	Ecuador, Napo	ROM 105305		JF448943
	Ecuador, Napo	ROM 105526		JF448944
	Ecuador, Napo	ROM 105524		JF448945
*Molossopsgriseiventer*	Antioquia		MACAU 005 19*	MACAU 005 19*
***Molossopsgriseiventer***	**Huila**	**ICN 25759**	**OR497272**	
	**Huila**	**ICN 25760**	**OR497273**	
*Eumopsauripendulus*		IP 9652809	JX444120	
				JF454657
*Cynomopsabrasus*		BDP 2178	CQ424038	
*Cynomopsplanirostris*				EF080314
*Tadaridateniotis*		NMP 48458	CQ424036	
				HM541963
*Nyctinomopsaurispinosus*		MSB 49749	KC747674	
*Nyctinomopslaticaudatus*				JF447290
